# Extracellular polysaccharide synthesis in a bloom-forming strain of *Microcystis aeruginosa*: implications for colonization and buoyancy

**DOI:** 10.1038/s41598-018-37398-6

**Published:** 2019-02-04

**Authors:** Meng Chen, Li-Li Tian, Chong-Yang Ren, Chun-Yang Xu, Yi-Ying Wang, Li Li

**Affiliations:** Shandong Provincial Key Laboratory of Water Pollution Control and Resource Reuse, School of Environmental Science and Engineering, Shandong University, Qingdao, China

## Abstract

*Microcystis*, the dominant species among cyanobacterial blooms, normally forms colonies under natural conditions but exists as single cells or paired cells in axenic laboratory cultures after long-term cultivation. Here, a bloom-forming *Microcystis aeruginosa* strain CHAOHU 1326 was studied because it presents a colonial morphology and grows on the water surface during axenic laboratory culturing. We first examined the morphological features of strain CHAOHU 1326 and three other unicellular *M. aeruginosa* strains FACHB-925, FACHB-940, and FACHB-975 cultured under the same conditions by scanning and transmission electron microscopy. Then, we compared the extracellular polysaccharide (EPS)-producing ability of colonial strain CHAOHU 1326 to that of the three unicellular *M. aeruginosa* strains, and found that strain CHAOHU 1326 produced a higher amount of EPS than the other strains during growth. Moreover, based on genome sequencing, multiple gene clusters implicated in EPS biosynthesis and a cluster of 12 genes predicted to be involved in gas vesicle synthesis in strain CHAOHU 1326 were detected. These predicted genes were all functional and expressed in *M. aeruginosa* CHAOHU 1326 as determined by reverse transcription PCR. These findings provide a physiological and genetic basis to better understand colony formation and buoyancy control during *M. aeruginosa* blooming.

## Introduction

The proliferation of cyanobacteria in eutrophic water bodies resulting in harmful algal blooms has occurred worldwide for decades^1,2^. Cyanobacterial blooms impair aquatic ecosystems and cause deterioration of water quality, thus seriously threatening water bodies throughout the world. With global climate changes, cyanobacterial blooms are predicted to expand and have thereby attracted increasing concern^3^. *Microcystis* is one of the most ubiquitous bloom-forming cyanobacteria in freshwater ecosystems^4^. Many *Microcystis* strains produce hepatotoxin microcystins, which pose a risk to humans when the cyanobacterial-blooming water serves drinking, fishery, and recreational needs^5^. There are multiple factors leading to cyanobacterial bloom formation. Environmental factors, such as nutrient loading, water temperature rises, and enhanced stratification, are regarded as the drivers of bloom formation^3^. Cyanobacteria cells own some competitive advantages against their opponents in aquatic ecosystems. For example, *Microcystis* has some adaptations that are favourable to survival in aquatic environments, such as buoyancy control, an annual growth cycle with a special storage strategy, efficient nitrogen uptake, and resistance to zooplankton grazing^1^. Considering the interaction between these factors, it is not surprising that there are repeated outbreaks of cyanobacterial blooms in some specific waterbodies. It is therefore an ongoing global challenge to control cyanobacterial blooms.

During cyanobacteria blooming, large colonial aggregates form a scum floating on the surface of water bodies^6^. Numerous studies have shown that extracellular polysaccharides (EPS) are involved in the colony formation of *Microcystis*^7–9^. EPS is the main component of the matrix embedding the proliferating cells and promoting colony formation. EPS is also involved in regulating buoyancy as the size of colonies positively correlates to the degree of buoyancy^10^. Therefore, insight into EPS synthesis in bloom-forming *Microcystis* may provide clues towards a better understanding of the mechanisms of cyanobacterial blooming. The morphology of *Microcystis aeruginosa* has been found to differ between natural environments compared with laboratory conditions. In natural water bodies, *M. aeruginosa* tends to form colonial aggregates, i.e., several tens to hundreds of cells aggregate in the mucilage^11^. Studies have illustrated that distinct colony morphotypes of *Microcystis* are shaped by the mucilage that embeds the cells^12^. However, *Microcystis* isolates always lose the ability to form colonies when cultivated in the laboratory under axenic conditions^13^. It is interesting that the amount of EPS produced by laboratory-cultured unicellular cells is considerably decreased compared with that produced by cells growing in colonies^14^.

In the present study, we found that the bloom-forming *M. aeruginosa* strain CHAOHU 1326 maintains a colonial morphology and grows on the water surface during laboratory culturing under axenic conditions. Strain CHAOHU 1326 was therefore a suitable target for examining colony formation and buoyancy control. The purpose of the present study was to examine the physiological and genetic basis for colony formation and buoyancy control in strain CHAOHU 1326. We first examined the morphological features of strain CHAOHU 1326. Then, we compared the EPS-producing ability of colonial strain CHAOHU 1326 to that of unicellular *M. aeruginosa* strains. We also characterized the soluble exopolysaccharide produced by strain CHAOHU 1326. Further, enzymes and proteins possibly involved in EPS biosynthesis and gas vesicle formation in strain CHAOHU 1326 were analysed based on genome sequencing and transcription analysis. The findings of this study will enhance our understanding of colony formation and buoyancy control during *M. aeruginosa* blooming.

## Results

### Phylogenetic analysis of the bloom-forming *M. aeruginosa* strain CHAOHU 1326

**Figure 1:**
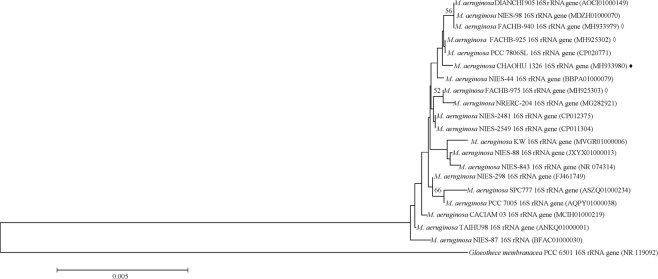
Neighbour-joining phylogenetic tree based on the 16S rRNA gene sequences of *M. aeruginosa* CHAOHU 1326 and other *M. aeruginosa* strains obtained from the GenBank database. Bootstrap values above 50% are shown at the branch nodes (1,000 replicates). *Gloeothece membranacea* PCC 6501 is used as outgroup. The scale bar represents 0.005 nucleotide substitutions per site. *M. aeruginosa* CHAOHU 1326 is indicated by the solid diamond (♦). Strains FACHB-925, FACHB-940, and FACHB-975 are marked by the empty diamond (◊).

Phylogenetic analysis of the 16S rRNA gene sequences of *M. aeruginosa* CHAOHU 1326 and nineteen other *M. aeruginosa* strains was performed and the results are shown in Fig. 1. From the phylogenetic tree, strain CHAOHU 1326 was closely related to the previously reported *M. aeruginosa* strains DIANCHI 905, NIES-98, and PCC 7806SL. Among them, strain PCC 7806SL is a representative strain for microcystin production^15^, in contrast, NIES-98 is a non-microcystin-producing strain^16^, and strain DIANCHI 905 is a bloom-forming strain^17^. Strain CHAOHU 1326 also shared substantial similarity to other two strains in the present study, i.e., strains FACHB-925 and FACHB-940.

### Morphological characteristics of *M. aeruginosa* CHAOHU 1326

**Figure 2:**
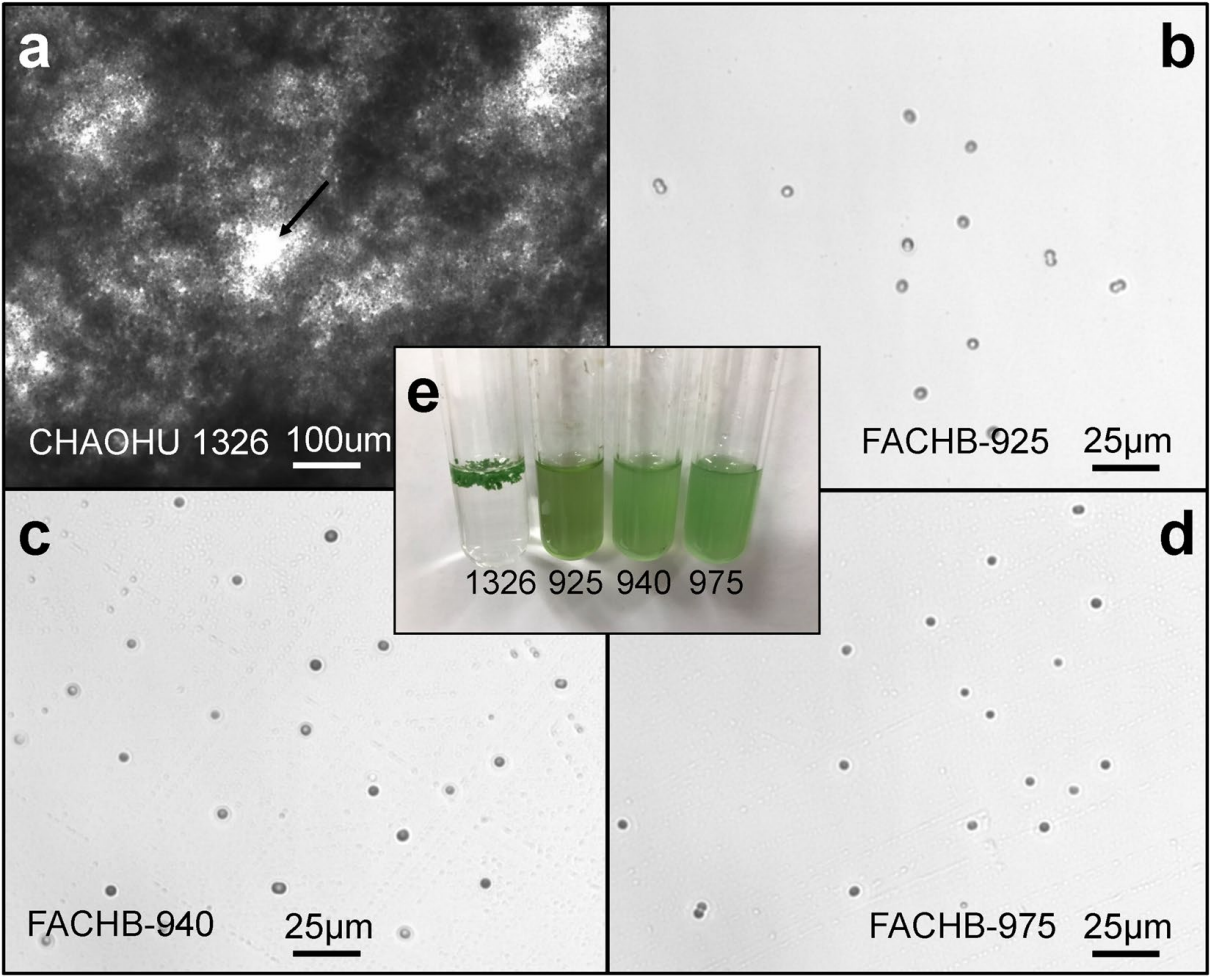
Cultures of *M. aeruginosa* strains grown to early exponential phase in BG11 medium. Panels a–d show the cells of *M. aeruginosa* strains CHAOHU 1326, FACHB-925, FACHB-940, and FACHB-975, respectively. The macroscopic appearance of the *M. aeruginosa* cultures is shown in panel e, and the strains are marked by the numbers corresponding to their strain names. The small hole between colonies of strain CHAOHU 1326 is marked with an arrow in panel a.

Photomicrographs of the four *M. aeruginosa* strains, namely CHAOHU 1326, FACHB-925, FACHB-940, and FACHB-975, at mid-logarithmic growth phase in BG11 medium are shown in Fig. 2. The cells of strain CHAOHU 1326 formed colonies during growth (Fig. 2a). By contrast, the cells of strains FACHB-925, FACHB-940, and FACHB-975, existed mainly as single cells, plus a few binate cells (Fig. 2b–d). As shown in Fig. 2e, strain CHAOHU 1326 was distinct in that it formed thin layers of aggregated colonies on the surface of the BG11 medium, whereas strains FACHB-925, FACHB-940, and FACHB-975 remained unicellular and well-distributed in the medium during growth. More specifically for strain CHAOHU 1326, the spherical cells became densely agglomerated. In the early stages of growth, the cells were smooth in appearance and formed small colonies without an internal hollow, whereas in the later stages of growth, colonies became diffuse and irregular in shape with small holes (Fig. 2a).

**Figure 3:**
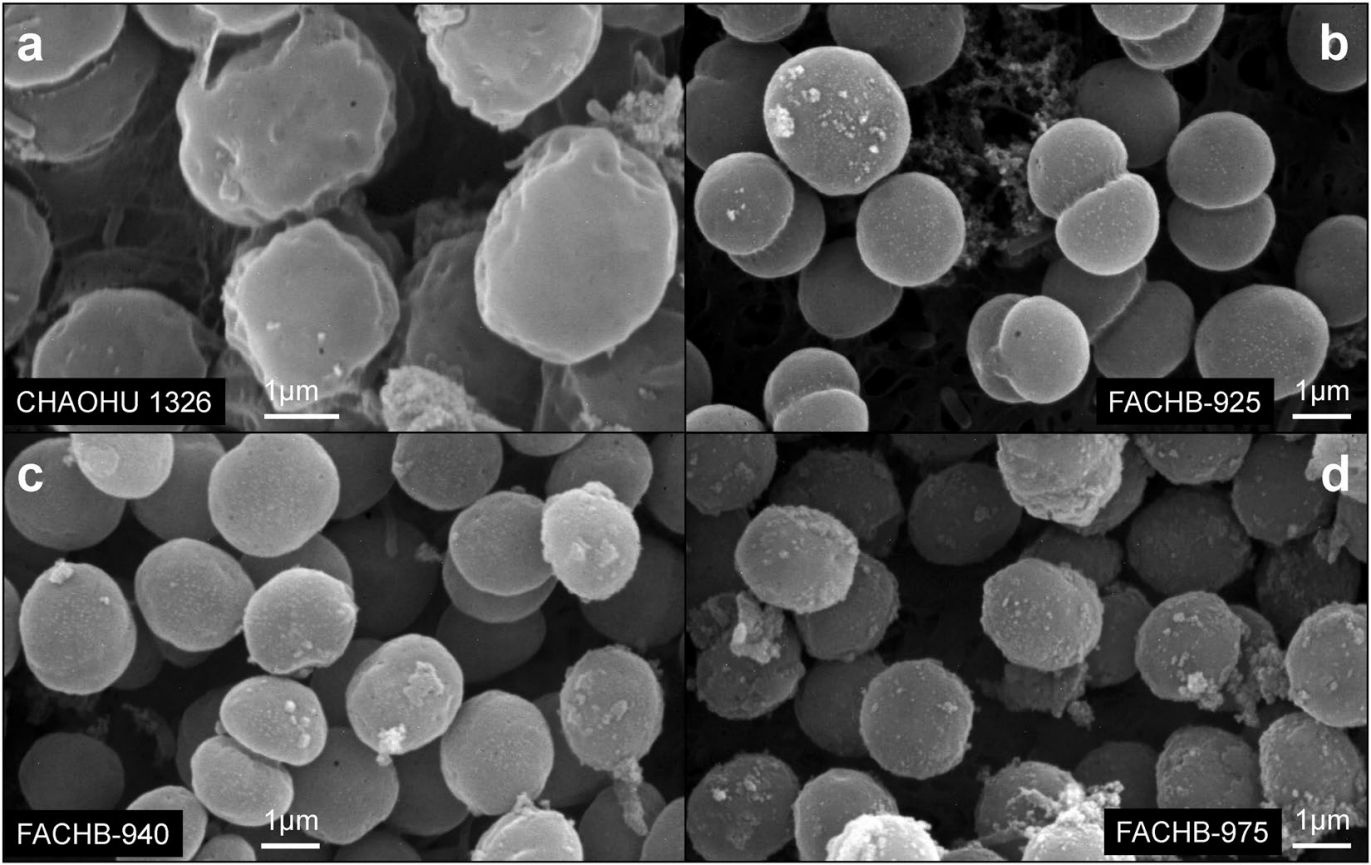
SEM images of the four *M. aeruginosa* strains grown to mid-exponential phase in BG11 medium, including *M. aeruginosa* CHAOHU 1326 (**a**), *M. aeruginosa* FACHB-925 (**b**), *M. aeruginosa* FACHB-940 (**c**), and *M. aeruginosa* FACHB-975 (**d**).

The morphologies of the four *M. aeruginosa* strains in mid-logarithmic growth phase in BG11 medium was observed, as shown by representative scanning electromicroscopic images (Fig. 3). The cells of strain CHAOHU 1326 appeared irregular in shape and were encased by a thick extracellular layer, resulting in the large cell agglomerates evident in Fig. 3a. By contrast, the cells of strains FACHB-925, FACHB-940, and FACHB-975 appeared as relatively small, distinct, regular shaped cells (Fig. 3b–d).

**Figure 4:**
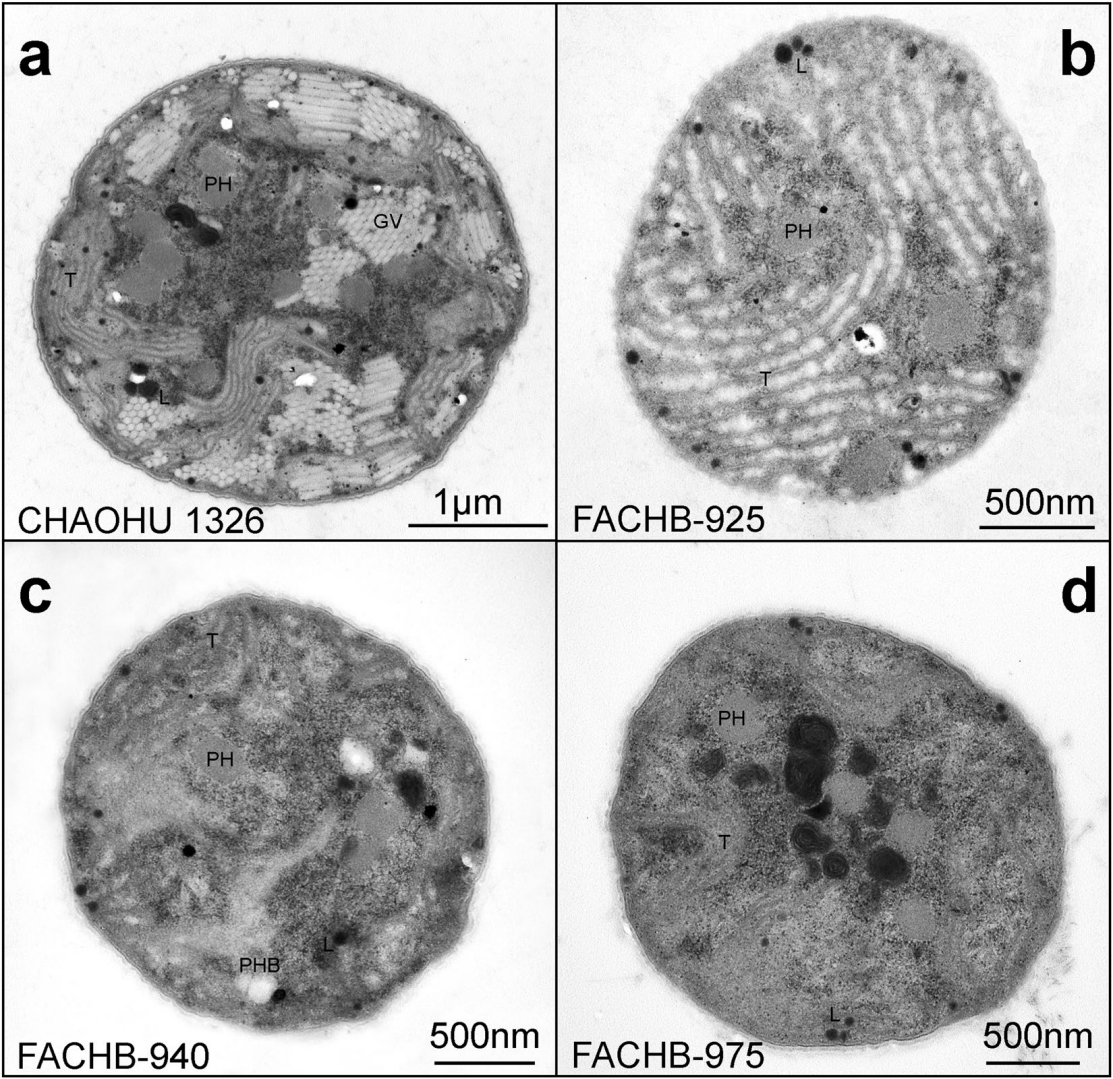
TEM images of the four *M. aeruginosa* strains at mid-exponential growth phase in BG11 medium, including strains CHAOHU 1326 (**a**), FACHB-925 (**b**), FACHB-940 (**c**), and FACHB-975 (**d**). Poly-beta-hydroxybutyrate (PHB), poly granules (PH), lipid droplets (L), thylakoids (T), and gas vesicles (GV) are indicated.

The sections of the four *M. aeruginosa* strains showed ultrastructural components such as thylakoids, gas vesicles, granules, lipid droplets, and the plasma membrane of the cells (Fig. 4). Massive gas vesicles were regularly observed between the thylakoids of CHAOHU 1326 cells that were not detected in the other *M. aeruginosa* strains. For example, the cells of strain FACHB-925 were full of thylakoids but no gas vesicles were observed.

Generally, the cell size of strain CHAOHU 1326 was larger than that of the unicellular-growing strains FACHB-925, FACHB-940, and FACHB-975. The cells of strain CHAOHU 1326 were with a diameter of 4.14 ± 0.4 μm, almost twice that of the other strains. FACHB-925, FACHB-940, and FACHB-975 were 1.83 ± 0.2 μm, 2.36 ± 0.2 μm, and 1.76 ± 0.2 μm in diameter, respectively (p < 0.05).

### EPS synthesis by *M. aeruginosa* CHAOHU 1326

**Figure 5:**
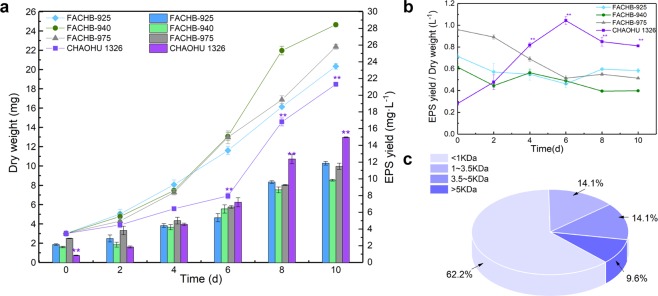
Soluble EPS produced by the *M. aeruginosa* strains during growth in BG11 medium. The EPS yields are displayed by the columns and the growth curves of different strains are displayed by lines in panel a. The ratios of EPS yield to the dry cell weight of the different strains are shown in panel b. The molecular weight distribution of soluble EPS produced by *M. aeruginosa* CHAOHU 1326 is shown in panel c. Error bars represent standard deviation in at least triplicate. A statistical difference of p < 0.01 is marked with asterisks (**).

The dry weights and EPS yields of the four *M. aeruginosa* strains during growth are shown in Fig. 5. Under the same initial inoculum dosage (3 mg dry weight), the growth potential of *M. aeruginosa* strains CHAOHU 1326, FACHB-925, FACHB-940, and FACHB-975 was different throughout the incubation time. Regarding the initial growth rate and biomass, strain CHAOHU 1326 was inferior compared with the other unicellular morphology strains (Fig. 5a). However, the yield of EPS by strain CHAOHU 1326 increased with incubation time (Fig. 5a), and the amount of EPS produced by *M. aeruginosa* CHAOHU 1326 exceeded that of other strains after 4 d significantly (Fig. 5b). The results indicated that strain CHAOHU 1326 produced a significantly higher amount of EPS than other unicellular morphology strains during growth.

The molecular weight distribution of EPS produced by *M. aeruginosa* CHAOHU 1326 was investigated. As shown in Fig. 5c, carbohydrates with a molecular weight of less than 1 kDa were dominant and accounted for 62.2% of the total soluble EPS, while carbohydrates with a molecular weight of more than 5 kDa accounted for less than 10% of the total soluble EPS.

### Genome basis for polysaccharide synthesis in *M. aeruginosa* CHAOHU 1326

**Figure 6:**
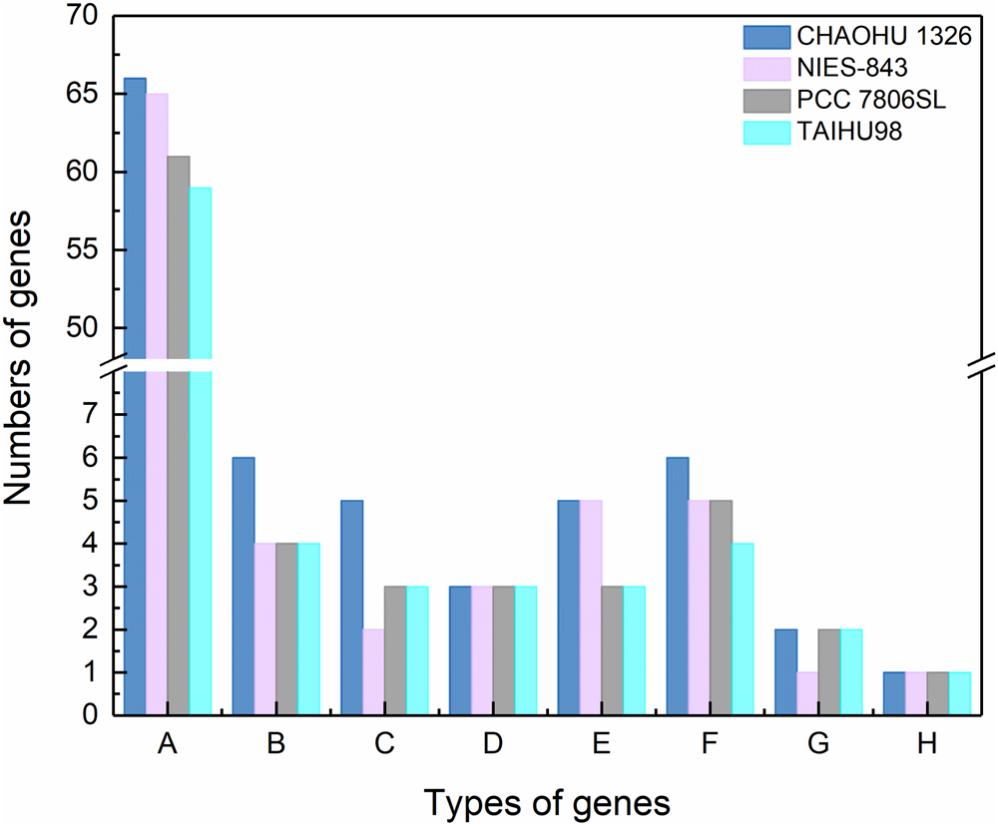
Types and numbers of genes encoding enzymes and proteins involved in extracellular polysaccharide biosynthesis and export in *M. aeruginosa* strains CHAOHU 1326, NIES-843, PCC 7806SL and TAIHU98. A: glycosyltransferases; B: isomerases; C: epimerases; D: phosphoglucomutases; E: glucose dehydrogenases; F: polysaccharide biosynthesis proteins; G: polysaccharide export proteins; H: bifunctional protein GlmU.

In cyanobacteria, there are a group of genes participating in exopolysaccharide biosynthesis and export^12^. Genome sequence analysis revealed that strain CHAOHU 1326 carries multiple gene clusters implicated in EPS synthesis and export, such as the phosphorylation of glucose and other monosaccharides, the formation and transformation of sugar nucleotides, the synthesis and modification of EPS repeating units, polymerization, and export (Fig. 6). Specifically, compared with other *M. aeruginosa* strains, including NIES-843^12^, PCC7806SL^18^, and TAIHU98^19^, the genome of strain CHAOHU 1326 contains more genes encoding glycosyl transferase, isomerases, epimerases, and polysaccharide biosynthesis proteins (Fig. 6). For example, six isomerases genes have been detected in the genome of strain CHAOHU 1326, encoding mannose-6-phosphate isomerase, glucose-6-phosphate isomerase, and ribose-5-phosphate isomerase, which catalyse a series of the reversible isomerization reactions^20,21^. Additionally, there are five genes encoding epimerases present in the genome of strain CHAOHU 1326, including UDP-glucose 4-epimerase and UDP-N-acetylglucosamine 2-epimerase, which interconverts UDP-glucose and UDP-galactose, and converts UDP-N-acetylglucosamine into UDP-N-acetylmannosamine, respectively^22,23^.

**Figure 7:**
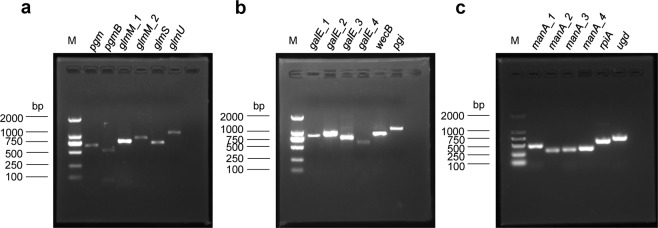
Transcriptional activity of genes involved in polysaccharide synthesis in *M. aeruginosa* CHAOHU 1326. The reverse transcription PCR products for *pgm* encoding an alpha-D-glucose phosphate-specific phosphoglucomutase, *pgmB* encoding a beta-phosphoglucomutase, *glmM_1* encoding a phosphoglucosamine mutase, *glmM_2* encoding a phosphoglucosamine mutase, *glmS* encoding a glutamine-fructose-6-phosphate aminotransferase, and *glmU* encoding bifunctional protein GlmU, are shown in panel a. Amplicons for *galE_1* encoding a UDP-glucose 4-epimerase, *galE_2* encoding a UDP-glucose 4-epimerase, *galE_3* encoding a UDP-glucose 4-epimerase, *galE_4* encoding a UDP-glucose 4-epimerase, *wecB* encoding a UDP-N-acetylglucosamine 2-epimerase, and *pgi* encoding a glucose-6-phosphate isomerase, are shown in panel b. Amplicons for *manA_1* encoding a mannose-6-phosphate isomerase, *manA_2* encoding a mannose-6-phosphate isomerase, *manA_3* encoding a mannose-6-phosphate isomerase, *manA_4* encoding a mannose-6-phosphate isomerase, *rpiA* encoding a ribose-5-phosphate isomerase, and *ugd* encoding a UDP-glucose 6-dehydrogenase are shown in panel c. (M) is a corresponding DNA molecular mass standard.

The predicted genes involved in EPS synthesis (Supplementary Table S1) were subjected to transcriptional activity tests. As shown in Fig. 7, the 18 genes selected all showed transcriptional activity in strain CHAOHU 1326, which encode phosphoglucomutase^24^, phosphoglucosamine mutase^25^, UDP-glucose 4-epimerase^22^, UDP-N-acetylglucosamine 2-epimerase^23^, glucose-6-phosphate isomerase^20^, mannose-6-phosphate isomerase^21^, glutamine-fructose-6-phosphate aminotransferase^26^, bifunctional protein GlmU^26^, and UDP-glucose 6-dehydrogenase^27^. No corresponding amplicons were observed in the negative control when purified RNA was used as the template (Supplementary Fig. S1). The results indicated that these EPS synthesis genes are functional in strain CHAOHU 1326. Meanwhile, it was noticed that some of the genes were non-transcribed in other *M. aeruginosa* strains, such as FACHB-925 and FACHB-940 (Supplementary Fig. S2). The function of these genes in different *M. aeruginosa* strains needs further study.

### Genome basis for gas vesicle synthesis in *M. aeruginosa* CHAOHU 1326

**Figure 8:**
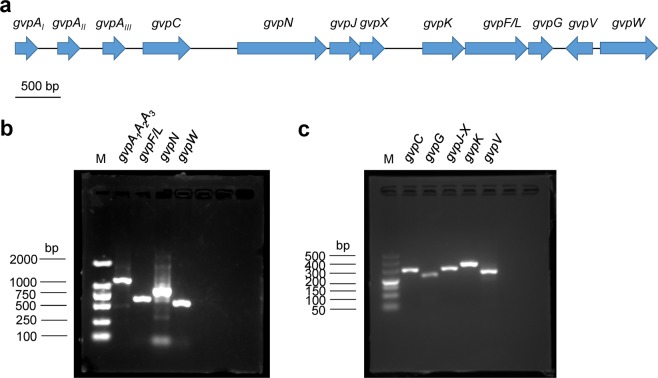
Organization and transcriptional analysis of genes involved in gas vesicle synthesis of *M. aeruginosa* CHAOHU 1326. The organization of the *gvp* gene cluster in *M. aeruginosa* CHAOHU 1326 is shown in panel a. The RT-PCR products of *gvpA*, *gvpF/L*, *gvpN*, and *gvpW* are shown in panel b, and the amplicons of *gvpC*, *gvpG*, *gvpJ-X*, *gvpK*, and *gvpV* are shown in panel c. (M) is a corresponding DNA molecular mass standard.

The genome sequence of *M. aeruginosa* CHAOHU 1326 revealed a cluster of twelve genes predicted to be involved in gas vesicle synthesis, including *gvpW*, *gvpV*, *gvpG*, *gvpF/L*, *gvpK*, *gvpX*, *gvpJ*, *gvpN*, *gvpC*, and three copies of *gvpA* (Fig. 8a). All of these genes were in the same transcriptional direction except *gvpV*. The deduced amino acid sequences of the three copies of *gvpA* (*gvpA*_1_, *gvpA*_2_, and *gvpA*_3_) were identical, thereby indicating that they encoded the same major structural protein GvpA. The deduced amino acid sequence of GvpA of strain CHAOHU 1326 was identical to that of GvpAII or GvpAIII (WP_002735503.1) in *M. aeruginosa* strain PCC 7806SL. Generally, the deduced amino acid sequences of all of the *gvp* genes of strain CHAOHU 1326 showed high identities (87–100%) to that of strain PCC 7806SL.

Expression of the *gvp* genes in strain CHAOHU 1326 was examined by reverse transcription PCR (Fig. 8b,c). Amplification products corresponding to *gvpW*, *gvpV*, *gvpG*, *gvpF/L*, *gvpK*, *gvpJ-X*, *gvpN*, *gvpC*, and *gvpA* were obtained using cDNA as template. No corresponding amplicons were detected in the negative control when purified RNA was used as the template (Supplementary Fig. S3). The results indicated that all of the predicted *gvp* genes were expressed in *M. aeruginosa* CHAOHU 1326 in late exponential growth phase.

## Discussion

The formation of large colonial aggregates plays an important role in cyanobacteria blooming^9^. Commonly, large colonies migrate quickly and float to the surface to form blooms^28,29^. Colonial morphology is advantageous in resisting the damage caused by adverse factors on *Microcystis* cells, such as heavy metal stress and high-light inhibition^30,31^. Colony formation is also advantageous in resisting predation by zooplankton in aqueous ecosystems^8^. It has been reported that *M. aeruginosa* strains commonly form large colonial aggregates in natural aquatic environments but tend to disaggregate to single cells under laboratory conditions^13,32^. *M. aeruginosa* strain CHAOHU 1326 is an exception showing colonial growth in BG11 medium during long-term laboratory cultivation. A previous study demonstrated that the colonial cells and their disaggregated counterparts could be divided into two morphotypes according to distinguishing morphological features^13^. It was revealed that the phenotypic variations from an aggregated form into a disaggregated form were not accompanied by genotypic variations^13^. The phenotypic plasticity of *Microcystis* species provides an adaptive advantage in their environmental niche. It was suggested that the variation in EPS synthesis may play a role in the aggregation state of *M. aeruginosa* cells. Although the involvement of EPS in colony formation is generally accepted for *Microcystis* species, the reason for colony disaggregation during laboratory culture was not clear. A previous study demonstrated that a high EPS content and colony formation were associated with a low specific growth rate^33^. The authors proposed that the phenotypic variation during growth under axenic laboratory cultures may be due to the specific growth rate in laboratory cultures exceeding that under natural conditions^33^. In the present study, strain CHAOHU 1326 was shown to produce EPS at a high rate whilst maintaining a relatively slow growth rate compared with other low EPS-producing strains. In such low EPS-producing strains, a high growth rate is required to maintain a sufficient respiration rate for rapid cell division and consequently, EPS production is reduced. In strain CHAOHU 1326, high EPS production negatively influenced the growth rate. The invariant colonial morphology of strain CHAOHU 1326 seemed to benefit from high EPS production in coordination with low growth rate.

Compared with the disaggregated growth of strains under laboratory conditions, strain CHAOHU 1326 produced a high amount of EPS as a result of an abundance of polysaccharide synthesis genes. Genome sequence analysis combined with transcription analysis indicated a series of functional genes for EPS synthesis in strain CHAOHU 1326, which were involved in the phosphorylation of monosaccharides, the formation and transformation of sugar nucleotides and polysaccharide synthesis, the modification of EPS repeating units, and the polymerization and export of polysaccharides. Cyanobacterial exopolysaccharides consist of repeating units built from monosaccharides that result in molecules several hundred kDa in size^12^. Although only a few EPS structures have been proposed to date, the majority of EPS structures analysed in cyanobacteria were characterized as being of high molecular weight. In the present study, the main components in the EPS of strain CHAOHU 1326 were molecules of less than 1 kDa. The effect of EPS of low molecule weight on the colony forming ability of strain CHAOHU 1326 remains unclear.

During blooming, colonial cyanobacteria also benefit from their strong buoyancy. Gas vesicles provide buoyancy to *Microcystis* species and it was proposed that *Microcystis* cells must accumulate sufficient gas vesicles to support their buoyancy^5^. As a result of inorganic carbon diffusion being limited in large colonies below the water surface, the internal cells of colonial aggregates maintain a high degree of buoyancy and form a surface scum, allowing the cells access to atmospheric carbon dioxide^1^. The benefit of buoyancy is that it lifts *Microcystis* cells closer to the water surface where the higher irradiance supports a higher rate of photosynthesis. Efficient photosynthesis promotes the production of carbohydrate in cells. In *Microcystis* cells, carbon also plays a role in the regulation of buoyancy. Polysaccharide synthesis and accumulation is one of the strategies employed by the cells to control buoyancy. On the one hand, polysaccharide counteracts the buoyancy provided by gas vesicles if it is stored as polyglucose granules in cells. On the other hand, it is respired and converted into a less dense protein^5^, which promotes the synthesis of gas vesicle protein. In this scenario, EPS synthesis contributes to colony formation in *Microcystis* species, which ensuring that the buoyant cyanobacteria float up to the surface of the water. According to Stokes equation, the floating velocity of a sphere is proportional to the square of its radius. Thus, the floating velocity of cyanobacterial colonies is strongly dependent on the colony size^5^. In this sense, EPS synthesis is also a strategy to control buoyancy.

In the present study, we demonstrated that EPS synthesis and buoyancy are in fine balance in the cells of *M. aeruginosa* strain CHAOHU 1326. Genome sequence analysis revealed not only abundant EPS synthesis genes but also a complete set of gas vesicle genes. Specifically, the genome contains three copies of *gvpA*, which encode the same small hydrophobic protein GvpA, the main constituent of gas vesicles. Gas vesicle genes distribute ubiquitously in *M. aeruginosa* genomes. According to the genome sequences of twenty-three *M. aeruginosa* strains available in GenBank, there are ten distinct *gvp* genes (A, C, N, J, X, K, F/L, G, V, and W) present in different strains (Supplementary Table S2). The product of *gvpA* is an obligatory component of gas vesicles, while other proteins in the gas vesicle gene cluster either provide additional strength by binding to the exterior surface of the vesicle wall or appear to have roles in the assembly process^34^. As shown in Supplementary Table S2, *gvpA* gene is present in two or three copies in most of *M. aeruginosa* genomes. Since a complete copy of *gvpA* is essential for GvpA protein and of a gas vesicle structure, only two of twenty-three strains lack of *gvpA* gene. It is noted that eight strains carry three copies of *gvpA*, including strain CHAOHU 1326. Multiple copies of *gvpA* in *M. aeruginosa* genome is thought to permit fast synthesis of large amounts of the major structural GvpA with minimal participation of the transcriptional machinery^35^. As the root of gas vesicle formation, the mechanisms of buoyancy regulation in *M. aeruginosa* may involve modulation of gas vesicle genes expression^36^. Studies have demonstrated that the expression of *gvp* genes was regulated at the post-transcriptional level under different environment conditions, such as pH, the sensitiveness to which was different for different strains^37^. As proposed by Mlouka *et al*.^35^, *M. aeruginosa* cells in laboratory culture were provided with an environment in which ample nutrient availability (e.g., that of CO_2_ and light irradiance) rendered a biased selection towards gas vesicle-deficient mutants. Regarding the buoyancy performance of different strains, it is unclear whether mutation or transcriptional regulation of *gvp* genes plays a role in the specific environment condition. Recent studies have implicated that buoyancy of cyanobacteria colonies could be driven by oxygen bubbles on or within the mucilage of the colonies^38–40^, which suggested a broad understanding of buoyancy regulation mechanism besides intracellular changes in carbohydrates or gas vesicles. Although the genome sequence of strain CHAOHU 1326 has provided clues as to the colonial growth and buoyancy of this strain, further studies are needed regarding the regulation of the genes involved in polysaccharide synthesis and gas vesicle synthesis.

## Methods

### *M. aeruginosa* strains and cyanobacteria culture conditions

*M. aeruginosa* CHAOHU 1326 (FACHB-1326), as well as other *M. aeruginosa* strains including FACHB-925, FACHB-940, and FACHB-975, were all obtained from the Freshwater Algae Culture Collection of the Institute of Hydrobiology (FACHB), Chinese Academy of Sciences (Wuhan, China). *M. aeruginosa* CHAOHU 1326 is an isolate from Chaohu Lake (31.95 N, 117.87 E, Hefei, China). All *M. aeruginosa* strains were batch-cultured in triplicate and maintained in 300 ml Erlenmeyer flasks containing 100 ml of sterilized Blue-Green medium (BG-11 medium)^41^. The initial pH of the medium was adjusted to 7.2. *M. aeruginosa* strains were cultured at 25 °C under a 40 μmol photon m^−2^ s^−1^ illumination intensity with a photoperiod of 12:12 (light: dark). The flasks were artificially shaken three times daily to maintain culture homogeneity.

### Phylogenetic analysis

A concatenated dataset of partial 16S rRNA gene sequences downloaded from the National Center for Biotechnology Information (NCBI) was used to construct a phylogenetic tree, including the sequences of *M. aeruginosa* CHAOHU 1326, FACHB-925, FACHB-940, FACHB-975 and other cyanobacterial strains. Sequences were aligned in the clustalw implementation and then the phylogenetic and molecular evolutionary analyses were conducted using MEGA version 7 software^42^. The evolutionary tree was inferred using the neighbour-joining method with 1,000 bootstrap replications. The evolutionary distances were computed using the maximum composite likelihood method and the units were the number of base substitutions per site. The analysis involved 21 nucleotide sequences. All positions containing gaps and missing data were eliminated. There were a total of 1295 positions in the final dataset.

### Morphological observations

The morphologies of different *M. aeruginosa* strains during growth at mid-logarithmic phase were observed using an inverted fluorescence microscope (Ti-s, Nikon, Japan) and were displayed using the NIS-Elements D 4.20.00 software. Cells of *M. aeruginosa* strains (about 10^6^ cells/ml) at mid-logarithmic growth phase were also observed by scanning electron microscopy (SEM) and transmission electron microscopy (TEM). Samples for SEM observation were prepared as follows: 2 ml of sample was washed three times with 0.1 M phosphate-buffered solution (PBS; pH 7.2), fixed in 2.5% glutaraldehyde at 4 °C for 24 h, then rinsed with 0.1 M PBS buffer (pH 7.2). Specimens were then dehydrated using an acetonitrile series three times for 10 min at each stage (30%, 50%, 70%, 80%, and 100%) and dried with a CO_2_ Critical-Point Dryer (XD-1, Eiko, Japan). Finally, specimens were sputter-coated with platinum by an ion spraying platinum plating instrument (IB-3, Eiko, Japan) and examined by SEM using a JSM-840 scanning electron microscope (JEOL Ltd., Japan). For TEM observation, samples were fixed with 3% glutaraldehyde in cacodylate buffer (0.1 M) at 4 °C for 4 h, then post-fixated in 1% osmium-tetroxide PBS for 2 h. After a second wash, the samples were dehydrated in a graded ethanol series with ethanol concentrations of 30%, 50%, 70%, 80%, 90%, and 100% before being embedded in Epon resin. Finally, ultrathin sections of approximately 75 nm were prepared, stained with uranyl acetate and lead citrate for 15 min each, and then examined by TEM using a JEM-1200EX transmission electron microscope (JEOL Ltd., Japan). To compare the size of different *M. aeruginosa* strains, thirty measurements of each strain diameter were performed on individual cell.

### Soluble EPS analysis

The growth of *M. aeruginosa* strains was determined every two days by measuring the dry cell weight. The *M. aeruginosa* liquid culture samples (100 ml) were centrifuged at 7,000 × *g* for 10 min. Then cells were harvested, washed three times with distilled water, and finally dried at 105 °C to a constant weight for measuring the dry weight. At the same time, the supernatants were collected and concentrated to about 40 ml by a rotary evaporator. Then powder active carbon was added for depigmentation and was removed thereafter by passing through a 0.45-µm filtration membrane. The clarified liquor was mixed with 4 times the volume of 95% ethanol and incubated overnight at 4 °C for EPS precipitation. The precipitate was concentrated by evaporation and resuspended with distilled water, and this was repeated twice to completely remove the ethanol. Then the EPS collection was dried at 60 °C for dry weight measurement. The prepared EPS was dissolved in distilled water (0.01%, w/v) as a stock solution for further tests. The polysaccharide solution was then applied to a dialysis membrane for the separation of 1, 3.5, and 5 kDa molecule (Sangon Biotech Co., Ltd., Shanghai). The concentration of each of the dialyzed fractions was determined by the phenol–sulfuric acid method^43^, using glucose as the standard.

### Genome sequencing and analysis genes of polysaccharide synthesis, export and gas vesicle synthesis

The genome sequencing of *M. aeruginosa* strain CHAOHU 1326 was performed using the Illumina massively parallel sequencing platform HiSeq. 4000 (Illumina, San Diego, CA, USA). A short-insert paired-end library with an average insert size of 350 bp was constructed. *De novo* genome assembly was carried out using SOAPdenovo software (version 2.04; http://soap.genomics.org.cn/soapdenovo.html)^44^. All reads were used for further gap closure by the GapCloser software (version 1.12). Annotation was performed using the NCBI Prokaryotic Genome Annotation Pipeline^45^ that utilizes GeneMarkS +^46^, BLASTn^47^, andaz tRNAscan-SE^48^. A whole genome BLAST search (E-value ≤ 10^−5^, minimal alignment length percentage ≥40%) was performed against five databases including the GenBank non-redundant protein database (NR; http://www.ncbi.nlm.nih.gov/ version 20150405), Kyoto Encyclopedia of Genes and Genomes database (KEGG; http://www.genome.jp/kegg/ version 59), COG database, Gene Ontology database (GO; http://geneontology.org/ version 20150405), and SwissProt database (Swiss-Prot; http://www.ebi.ac.uk/uniprot/ version 20150414).

### Transcription analysis

To determine the transcriptional activities of genes involved in polysaccharide synthesis and gas vesicle synthesis, total RNA was isolated from strains CHAOHU 1326, FACHB-925, and FACHB-940 at exponential growth phase using the RNeasy Mini Kit (Qiagen, Hilden, Germany). The isolated RNA was DNase-treated using RNase-free DNase Set (Qiagen, Hilden, Germany). The purified RNA was reverse transcribed to complementary DNA (cDNA) with random primers using the ReverAid™ First Strand cDNA Synthesis Kit (Fermentas, Burlington, Canada). Transcription of the target genes was detected using specific primers, as described in Supplementary Tables S3 and S4. These primers were designed using the NCBI Primer-BLAST tool based on the genome sequence of strain CHAOHU 1326.

### Nucleotide sequence accession number

The whole genome sequence of *M. aeruginosa* CHAOHU 1326 has been deposited in the DDBJ/ENA/GenBank database under accession number MOLZ00000000. The nucleotide sequences of 16S rRNA gene of strains CHAOHU 1326, FACHB-925, FACHB-940, and FACHB-975 in this study are available under GenBank accession numbers MH933980, MH925302, MH933979, and MH925303, respectively.

### Statistical analysis

At least independent triplicate experiments were conducted. Data were statistically analyzed using SPSS 24 software. Statistical significance was assessed by independent-samples *t*-tests or using one-way analysis of variance (ANOVA) followed by Duncan’s and Tukey post-hoc tests. In all the tests, p < 0.05 was considered as significant. All data are expressed as the means ± standard deviation.

## Supplementary information

Extracellular polysaccharide synthesis in a bloom-forming strain of <i>Microcystis aeruginosa</i>: implications for colonization and buoyancy

## Data Availability

All constructs and strains are available on request.
