# Supplementary material for: Extracellular polysaccharide synthesis in a bloom-forming strain of Microcystis aeruginosa: implications for colonization and buoyancy
Source: Sci Rep. 2019 Feb 4;9:1251. doi: 10.1038/s41598-018-37398-6 (PMC6362013; doi:10.1038/s41598-018-37398-6)
Supplement: Supplementary file 1 — Extracellular polysaccharide synthesis in a bloom-forming strain of Microcystis aeruginosa: implications for colonization and buoyancy [file 41598_2018_37398_MOESM1_ESM.pdf]

Extracellular polysaccharide synthesis in a bloom-forming strain of *Microcystis aeruginosa*: implications for colonization and buoyancy

Authors

Meng Chen, Li-Li Tian, Chong-Yang Ren, Chun-Yang Xu, Yi-Ying Wang, Li Li*

Affiliation

Shandong Provincial Key Laboratory of Water Pollution Control and Resource Reuse, School of Environmental Science and Engineering, Shandong University, Qingdao, China

*** Corresponding author**

E-mail: lili@sdu.edu.cn

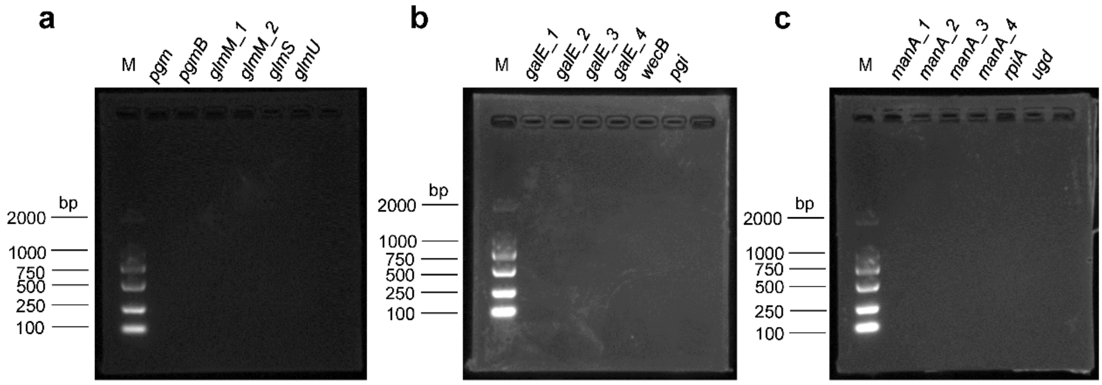

Figure S1. Negative control for transcriptional analysis of genes involved in polysaccharide synthesis in *M. aeruginosa* CHAOHU 1326, using purified RNA as the PCR template. The reverse transcription PCR products for *pgm* encoding an alpha-D-glucose phosphate-specific phosphoglucomutase, *pgmB* encoding a beta-phosphoglucomutase, *glmM_1* encoding a phosphoglucosamine mutase, *glmM_2* encoding a phosphoglucosamine mutase, *glmS* encoding a glutamine-fructose-6-phosphate aminotransferase, and *glmU* encoding bifunctional protein GlmU, are shown in panel a. Amplicons for *galE_1* encoding a UDP-glucose 4-epimerase, *galE_2* encoding a UDP-glucose 4-epimerase, *galE_3* encoding a UDP-glucose 4-epimerase, *galE_4* encoding a UDP-glucose 4-epimerase, *wecB* encoding a UDP-N-acetylglucosamine 2-epimerase, and *pgi* encoding a glucose-6-phosphate isomerase, are shown in panel b. Amplicons for *manA_1* encoding a mannose-6-phosphate isomerase, *manA_2* encoding a mannose-6-phosphate isomerase, *manA_3* encoding a mannose-6-phosphate isomerase, *manA_4* encoding a mannose-6-phosphate isomerase, *rpiA* encoding a ribose-5-phosphate isomerase, and *ugd* encoding a UDP-glucose 6-dehydrogenase are shown in panel c. (M) is a corresponding DNA molecular mass standard.

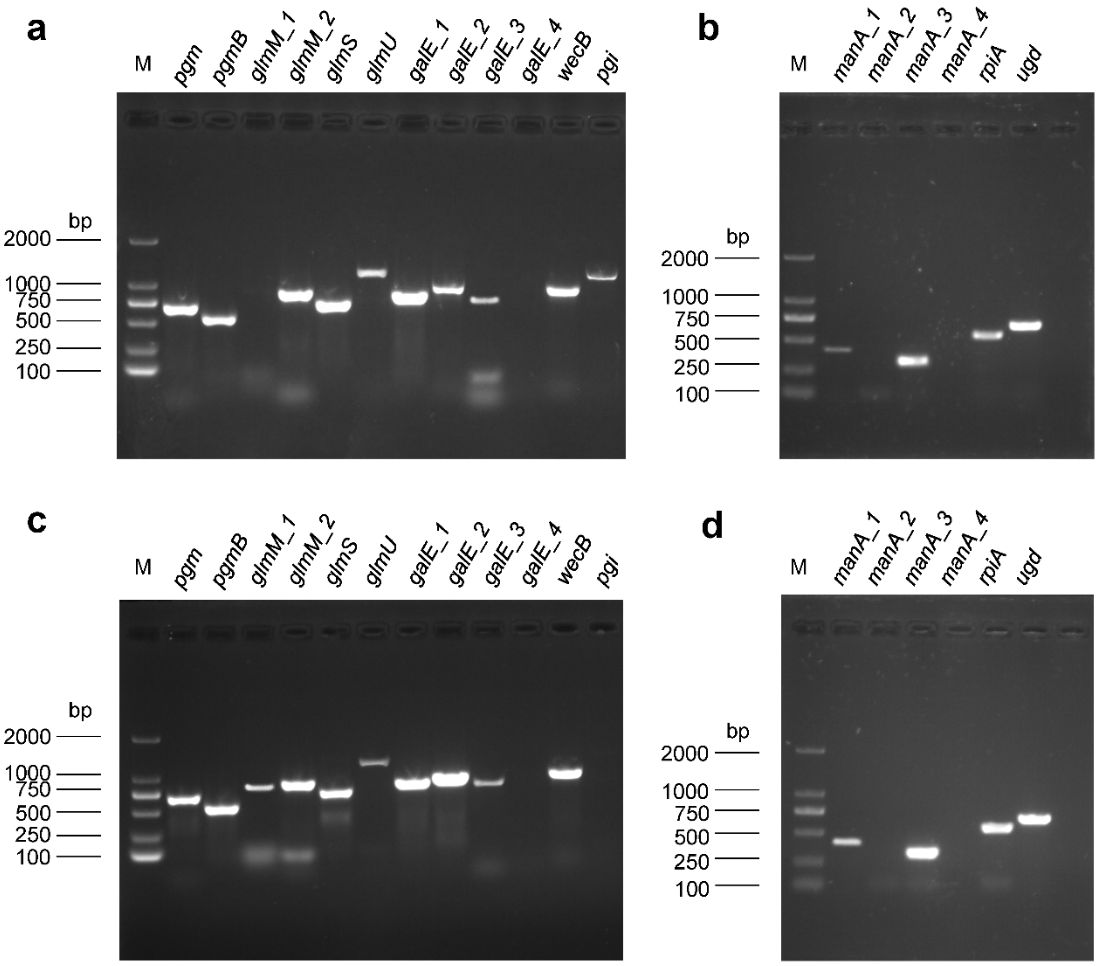

Figure S2. Transcriptional activity of genes involved in polysaccharide synthesis in *M. aeruginosa* strains FACHB-925 and FACHB-940. The reverse transcription PCR products for genes *pgm*, *pgmB*, *glmM_1*, *glmM_2*, *glmS*, *glmU*, *galE_1*, *galE_2*, *galE_3*, *galE_4*, *wecB*, and *pgi* of FACHB-925 are shown in panel a. Amplicons for genes *manA_1*, *manA_2*, *manA_3*, *manA_4*, *rpiA*, and *ugd* of FACHB-925 are shown in panel b. The reverse transcription PCR products for genes *pgm*, *pgmB*, *glmM_1*, *glmM_2*, *glmS*, *glmU*, *galE_1*, *galE_2*, *galE_3*, *galE_4*, *wecB*, and *pgi* of FACHB-940 are shown in panel c. Amplicons for genes *manA_1*, *manA_2*, *manA_3*, *manA_4*, *rpiA*, and *ugd* of FACHB-940 are shown in panel d. (M) is a corresponding DNA molecular mass standard.

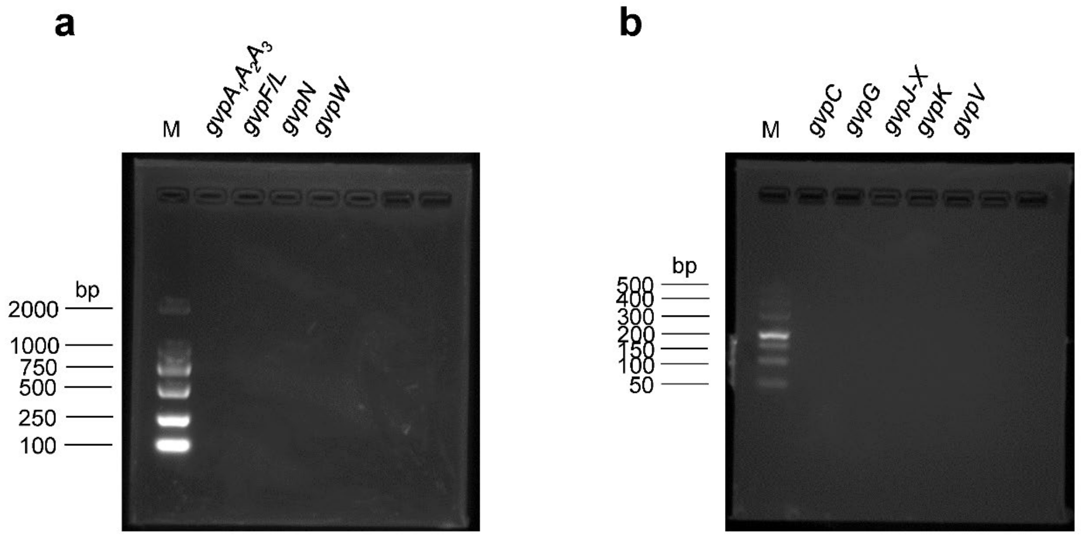

Figure S3. Negative control for transcriptional analysis of genes involved in gas vesicle synthesis of *M. aeruginosa* CHAOHU 1326, using purified RNA as the PCR template. The RT-PCR products of *gvpA*, *gvpF/L*, *gvpN*, and *gvpW* are shown in panel a, and the amplicons of *gvpC*, *gvpG*, *gvpJ-X*, *gvpK*, and *gvpV* are shown in panel b. (M) is a corresponding DNA molecular mass standard.

Description	Accession number	Coding product	Protein function	Reference
pgm	NZ_KV880541.1	alpha-D-glucose phosphate-specific phosphoglucomutase	catalyzes conversion of α-D-glucose 1-phosphate to α-D-glucose 6-phosphate	Chen, Z. *et al.*2016
pgmB	NZ_MOLZ01000240.1	beta-phosphoglucomutase	mediates conversion of β-D-glucose 1-phosphate to glucose 6-phosphate	Buckley *et al.* 2014
glmM_1	NZ_MOLZ01000010.1	phosphoglucosamine mutase	catalyzes conversion of glucosamine-6-phosphate to glucosamine-1-phosphate	Mehra-Chaudhary *et al.* 2011
glmM_2	NZ_MOLZ01000176.1	phosphoglucosamine mutase		
glmS	NZ_MOLZ01000290.1	Glutamine-fructose-6-phosphate aminotransferase	catalyzes conversion of fructose-6-phosphate to glucosamine-6-phosphate	
glmU	NZ_MOLZ01000066.1	bifunctional protein GlmU	catalyzes the N-acetylation of glucosamine 1-phosphate, and the uridylylation of *N*-acetyl-glucosamine 1-phosphate to form UDP-N-acetylglucosamine	Craggs *et al.* 2018
galE_1	NZ_MOLZ01000010.1	UDP-glucose 4-epimerase	interconverts UDP-glucose and UDP-galactose	Rösti *et al*. 2007
galE_2	NZ_MOLZ01000050.1	UDP-glucose 4-epimerase		
galE_3	NZ_MOLZ01000105.1	UDP-glucose 4-epimerase		
galE_4	NZ_MOLZ01000168.1	UDP-glucose 4-epimerase		
wecB	NZ_MOLZ01000035.1	UDP-N-acetylglucosamine 2-epimerase	converts UDP-N-acetylglucosamine into UDP-N-acetylmannosamine	Velloso *et al*. 2008
pgi	NZ_MOLZ01000236.1	glucose-6-phosphate isomerase	interconverts glucose-6-phosphate and fructose-6-phosphate	You *et al*. 2015
manA_1	NZ_KV880537.1	mannose-6-phosphate isomerase	catalyzes the reversible isomerization of D-mannose 6-phosphate and D-fructose 6-phosphate	Gowda *et al*. 2008
manA_2	NZ_MOLZ01000010.1	mannose-6-phosphate isomerase		
manA_3	NZ_MOLZ01000014.1	mannose-6-phosphate isomerase		
manA_4	NZ_MOLZ01000018.1	mannose-6-phosphate isomerase		
rpiA	NZ_MOLZ01000053.1	ribose-5-phosphate isomerase	catalyzes the inter-conversion of ribose 5-phosphate and ribulose 5-phosphate	Chen, Z. *et al.*2016
ugd	NZ_MOLZ01000038.1	UDP-glucose 6-dehydrogenase	catalyzes the conversion of UDP-glucose to UDP-glucuronic acid	Chen, Z. *et al.*2016

Table S1. Predicted genes involved in EPS biosynthesis in *M. aeruginosa* CHAOHU 1326.

Strains	Accession Number	*gvpA₁*	*gvpA₂*	*gvpA₃*	*gvpC*	*gvpN*	*gvpJ*	*gvpX*	*gvpK*	*gvpF/L*	*gvpG*	*gvpV*	*gvpW*
CHAOHU 1326	MOLZ00000000	+	+	+	+	+	+	+	+	+	+	+	+
DIANCHI 905	AOCI00000000	+	+	−	+	+	+	+	+	+	+	+	+
KW	MVGR00000000	+	+	−	+	+	+	+	+	+	+	+	+
NIES-44	BBPA00000000	+	+	−	+	+	+	+	+	+	+	+	+
NIES-87	BFAC00000000	+	+	−	+	+	+	+	+	+	+	+	+
NIES-88	JXYX00000000	+	+	−	−	+	+	+	+	+	+	+	+
NIES-98	MDZH00000000	+	+	+	+	+	+	+	+	+	+	+	+
NIES-843	AP009552	+	+	+	+	+	+	+	+	+	+	+	+
NIES-1211	BEIV00000000	+	+	−	+	+	+	+	+	+	+	+	+
NIES-2481	CP012375	+	+	−	+	+	+	+	+	+	+	+	+
NIES-2549	CP011304	+	−	−	+	+	+	+	+	+	+	+	+
PCC 7005	AQPY00000000	−	−	−	+	+	+	+	+	+	+	+	+
PCC 7806SL	CP020771	+	+	+	+	+	+	+	+	+	+	+	+
PCC 7941	CAIK00000000	+	+	−	+	+	+	+	+	+	+	+	+
PCC 9432	CAIH00000000	+	+	+	+	+	+	+	+	+	+	+	+
PCC 9701	CAIQ00000000	+	+	−	+	+	+	+	+	+	+	+	+
PCC 9717	CAII00000000	+	+	+	+	+	+	+	+	+	+	+	+
PCC 9806	CAIL00000000	+	+	−	+	+	+	+	+	+	+	+	+
PCC 9807	CAIM00000000	−	−	−	+	+	+	+	+	+	+	+	+
PCC 9808	CAIN00000000	+	−	−	+	+	+	+	+	+	+	+	+
PCC 9809	CAIO00000000	+	+	−	+	+	+	+	+	+	+	+	+
SPC777	ASZQ00000000	+	+	+	+	+	+	+	+	+	+	+	+
TAIHU98	ANKQ00000000	+	+	+	+	+	+	+	+	+	+	+	+

Table S2. List of genes involved in gas vesicles synthesis in *M. aeruginosa* strains.

Primers	Sequence (5'->3')	Target gene	Size (bp)
pgm-f	GCTGTACTAACAGCCAACGC	*pgm*	659
pgm-r	TGTAGGGCCTTTTGGGTGTC		
pgmB-f	GTCGTCAAGTGACCGAGGAG	*pgmB*	533
pgmB-r	TCGCTCCTTTTTATCCCCGA		
glmM1-f	TCCCGAATTAATCCAAAAATATGGT	*glmM_1*	773
glmM1-r	TTTCTGCCATGGCTGTCTCA		
glmM2-f	CGCGATCAAATCGGGACAAC	*glmM_2*	878
glmM2-r	CCCAGGCGAGAATTTCCTGA		
glmS-f	AGCAGTTGGCAGGTATTCCC	*glmS*	716
glmS-r	CAATAACCGCGCATCCCTTG		
glmU-f	AGGAACAGTTAGGCACTGGC	*glmU*	1075
glmU-r	AAGGCATTATCGGGGACATTTCT		
galE1-f	AGACGGCTATTGTGGTTGGG	*galE_1*	837
galE1-r	AATTCCCCCGATTGAGCAGG		
galE2-f	GGCTTTATTGCCAGCCACTT	*galE_2*	904
galE2-r	AGGGAGGTTGCCAATTCAGG		
galE3-f	CACGCCGAAATTATTAAGGATGTGT	*galE_3*	742
galE3-r	TTCTGGGACTTTCGATGACGG		
galE4-f	ACTGGTGTCGCGGGTTTTAT	*galE_4*	608
galE4-r	CCGTAGATGCCGATGGCTTT		
wecB-f	AGCTCCCGTTATTCGTGCTT	*wecB*	936
wecB-r	CCCGCTTCTGCTAAAATTTGCT		
pgi-f	AGGCATCGATCGCACCTTAG	*pgi*	1103
pgi-r	AGATTTGGAGCGTTTCCGGT		
manA1-f	AGCCAACTAACCAGCGATCA	*manA_1*	414
manA1-r	TGCGTCTAACTCCTCTGGGA		
manA2-f	TGGCACCATTTCAGCTTATTTCG	*manA_2*	301
manA2-r	AATACCAATTTAATCACCCCAGGAT		
manA3-f	TGTGAGGAATTCATCGCTGGT	*manA_3*	310
manA3-r	GCCAAGCAGGATCGACAATG		
manA4-f	CTCCCCTTATCCCCAAGACA	*manA_4*	350
manA4-r	CTGCATCATCCAAACGGTGAA		
rpiA-f	ATATCTGGGCGCTAGACTGC	*rpiA*	537
rpiA-r	ATCAGATCAGCAACCCCGAC		
ugd-f	GGTACACCAGCACTACCCAC	*ugd*	642
ugd-r	AGCTGACACATCTTTGGGGA		

Table S3. Primers used for transcriptional activity analysis of polysaccharide synthesis genes.

**Primers**	**Sequence (5'->3')**	**Target gene**	**Size (bp)**
gvpW-f	CAGGCTTTCTGCCAATGCAA	*gvpW*	514
gvpW-r	CCGTAGTCGAACCCGAAGTT		
gvpV-f	ATGACAACCACTCGTCCACC	*gvpV*	251
gvpV-r	GCTACACTAGGAGGGTTGGC		
gvpG-f	TCAGATTCGTTTTCTGCCTCT	*gvpG*	220
gvpG-r	TTACCGGTCCTATTGGGGGT		
gvpF/L-f	GCAAAGGTATAGGGTGCGGT	*gvpF/L*	575
gvpF/L-r	TTGGCATCCCGTCGCTATTT		
gvpK-f	CCTTCCACAACAACCCCTGT	*gvpK*	330
gvpK-r	TAGCCAAGCTGGTTTAGCCC		
gvpJ-f	TCACGCATCTGAGGGATGTG	*gvpJ-X*	277
gvpJ-r	TTCCGTTTCGATTGCCTCCA		
gvpN-f	ATCGGAAGGCTCCTCTTCCT	*gvpN*	801
gvpN-r	GCAGGAACGGGAAAAACCAC		
gvpC-f	TCTCTAGCCGTTTCGCTTCA	*gvpC*	276
gvpC-r	TGGAAAAGTTCCGCCAGGAG		
gvp$A_1A_2A_3$-f	CCCTACAGGGTTTTGCAGAT	*gvp*$A_1A_2A_3$	1042
gvp$A_1A_2A_3$-r	TTCCTACCAAAGAGACGCGA		

Table S4. Primers used for transcriptional activity analysis of gas vesicles genes.
